# Increase in *Bifidobacterium* is a characteristic of the difference in the salivary microbiota of pregnant and non-pregnant women

**DOI:** 10.1186/s12903-022-02293-0

**Published:** 2022-06-28

**Authors:** Satsuki Kato, Toshiyuki Nagasawa, Osamu Uehara, Shintaro Shimizu, Nodoka Sugiyama, Kozue Hasegawa-Nakamura, Kazuyuki Noguchi, Masayuki Hatae, Hiroshige Kakinoki, Yasushi Furuichi

**Affiliations:** 1 Division of Periodontology and Endodontology, Department of Oral Rehabilitation, School of Dentistry, Health Sciences University of Hokkaido, 1757 Kanazawa, Ishikari-Tobetsu, Hokkaido 0610293 Japan; 2 Division of Advanced Clinical Education, Department of Integrated Dental Education, School of Dentistry, Health Sciences University of Hokkaido, 1757 Kanazawa, Ishikari-Tobetsu, Hokkaido 0610293 Japan; 3 Division of Disease Control and Molecular Epidemiology, Department of Oral Growth and Development, School of Dentistry, Health Sciences University of Hokkaido, 1757 Kanazawa, Ishikari-Tobetsu, Hokkaido 0610293 Japan; 4 Department of Periodontology, Field of Oral and Maxillofacial Rehabilitation, Advanced Therapeutic Course, Kagoshima University Graduate School of Medical and Dental Sciences, 8-35-1 Sakuragaoka, Kagoshima, 8908532 Japan; 5 Kakinoki Hospital, 8-13 Kajiyamachi, Kagoshima, 8920846 Japan

**Keywords:** Pregnancy, Salivary microbiota, Estradiol, Progesterone, *Bifidobacterium*

## Abstract

**Background:**

The establishment of symbiotic microbiota in pregnant women is important for both the mother and her offspring. Little is known about the salivary symbiotic bacteria in pregnancy, and analysis of composition of microbiome (ANCOM) is useful to detect small differences in the number of bacteria. The aim of this study was to investigate the differences in the salivary bacteria between healthy pregnant and non-pregnant women using ANCOM.

**Methods:**

Unstimulated saliva samples were collected from 35 healthy pregnant women at 35 weeks gestation and 30 healthy non-pregnant women during menstruation. All participants underwent a periodontal examination. Estradiol and progesterone levels were examined by enzyme-linked immunosorbent assay. DNA extracted from the saliva was assessed by 16S ribosomal RNA amplicon sequencing and real-time PCR.

**Results:**

Salivary estradiol and progesterone levels were significantly increased in pregnant women. The alpha and beta diversities were higher in pregnant women than in non-pregnant women. The largest effect size difference noted when the microbiota of the pregnant and non-pregnant women were analyzed was that for *Bifidobacteriales*. Levels of *Bifidobacterium dentium,* but not of *Bifidobacterium adolescentis,* were significantly increased in pregnant women, and the levels were significantly correlated with progesterone concentration.

**Conclusion:**

The results suggest that *Bifidobacterium* and progesterone levels are elevated in the saliva of healthy pregnant women compared with non-pregnant women.

## Background

Plaque-induced gingivitis in women is modified by physiological alterations in the endocrine system, such as during puberty, menstruation, and pregnancy [1]. Gingival inflammation increases during pregnancy and is associated with an increase in female sex hormone levels [2–5]. Early investigations reported that estradiol and progesterone are nutrients for *Prevotella intermedia* (*P. intermedia*) and are correlated with pregnancy gingivitis, but recent reports based on molecular methods suggest that the subgingival microbiota is affected by gingival inflammation [6]. *Porphyromonas gingivalis* (*P. gingivalis*) significantly contributes to the worsening of gingival inflammation during pregnancy [4]. *Porphyromonas* *gingivalis* and *Aggregatibacter actinomycetemcomitans* (*A. actinomycetemcomitans*) are also associated with increased risk of preterm labor and preterm birth [7, 8]. Although abnormal pregnancy or pregnancy gingivitis may be associated with the proliferation of some pathogenic bacterial species, it is unclear whether pregnancy causes alterations in the symbiotic oral microbiota of healthy women.

Microbiota variation during pregnancy has been investigated using various techniques. Microbial-surveying techniques, such as 16S rRNA amplicon sequencing, are high-resolution methods that have provided excellent discrimination and detailed characterization of bacterial communities [9]. Saliva is a biological fluid secreted from the salivary glands into the oral cavity and contains bacteria shed from microbial communities adhering to various intraoral surfaces, including tooth surfaces, gingival crevices, tongue dorsum, and buccal mucosa. Also, saliva collection is easy, non-invasive, and usually takes a few minutes. However, few studies have used 16S rRNA amplicon sequencing to assess the saliva of pregnant women [10–12]. Lin et al. [11] performed 16S rRNA amplicon sequencing of supragingival plaque in 11 pregnant women and seven non-pregnant women and reported that *Neisseria, Porphyromonas,* and *Treponema* were more abundant in pregnant women. Crusell et al. [10] reported that the alpha diversity of the salivary microbiota decreases from the third trimester of pregnancy to the postpartum period. These results suggest that the salivary microbiota may change during pregnancy; however, the characteristic differences in the salivary microbiota between pregnant and non-pregnant women remain poorly understood.

We hypothesized that the levels of several symbiotic bacteria increase in the saliva of healthy pregnant women and that the symbiotic bacteria might not be the predominant species in terms of the number. We assume that the relatively small number of bacteria might be relevant to support a healthy pregnancy by excluding pathogenic bacteria that might otherwise increase during pregnancy. Analysis of composition of microbiome (ANCOM) is useful to detect small differences in the number of bacteria. The aim of this study was to investigate the differences in the salivary bacteria of healthy pregnant and non-pregnant women using ANCOM.

## Methods

### Study population

Thirty-five healthy pregnant women, who were at 35 weeks gestation, were recruited from the obstetrics clinic of Kagoshima City (Kakinoki Hospital) and the Health Sciences University of Hokkaido. Pregnancy outcomes, including age, height, weight, infant birth weight, and delivery complications, were recorded after delivery. Thirty healthy non-pregnant women also volunteered for this study. Non-pregnant women were examined during menstruation. All subjects agreed to participate in this study and signed a written informed consent form, and filled out a questionnaire, including smoking habits (never, former, or current). The subjects had no systemic disease (i.e., no diabetes, endocrine disorders, or hypertension) and did not use antibiotics or steroid hormones within the preceding 3 months. All participants were from medium-income households and had national insurance. Saliva sample collection and clinical examination were conducted on the same day. Prior to visiting the clinic, the subjects were asked to avoid eating, drinking, and brushing their teeth for 60 min prior to sampling. All pregnant women were instructed by obstetricians to not consume alcohol during pregnancy, and all non-pregnant women were instructed to not consume alcohol for at least one month before saliva sampling. Unstimulated saliva was collected immediately before the clinical examination. After saliva sample collection, the subjects underwent a periodontal examination, including assessment of the gingival index (GI), probing pocket depth (PPD), bleeding on probing (BOP), tooth mobility, periodontal epithelial surface area (PESA) [13], periodontal inflamed surface area (PISA) [13], and decayed, missing, and filled teeth (DMF). Clinical examination was conducted by two calibrated periodontal specialists (SK and KH-N). A healthy periodontal status was defined as having no probing attachment loss, probing depth ≤ 3 mm, and ≤ 10% BOP [14].

### Estradiol and progesterone assay

Saliva Collection Aid (Salimetrics, LLC, State College, PA, USA) was used to collect unstimulated saliva samples in a vial. After collection, the samples were stored at − 80 °C. On the day of the assay, the samples were centrifuged at 1500 g for 15 min to remove particulate matter, and the cleared samples were then subjected to enzyme-linked immunosorbent assay (ELISA) to detect estradiol (Salivary 17-Estradiol Enzyme Immunoassay Kit; Salimetrics) or progesterone (Salivary Progesterone Enzyme Immunoassay Kit; Salimetrics). All assay protocols are available from the kit manufacturers.

### Extraction of bacterial DNA from saliva samples

Unstimulated saliva was collected from each subject using OMNIgene™ Oral OM-505 (DNA Genotek Inc, Ottawa, ON, Canada) according to the protocol. For bacterial DNA extraction from the saliva, QIAamp® MinElute Virus Spin (Qiagen, Hilden, Germany) was used in accordance with the manufacturer’s protocol. DNA extracts were stored at − 20 °C and used for 16S rRNA amplicon sequencing or real-time PCR.

### Sequencing library preparation

The PCR reaction targeted the V3–V4 regions of the bacterial 16S rRNA gene. Sequencing libraries of the V3–V4 regions in the saliva samples were prepared according to the 16S Metagenomic Sequencing Library preparation instructions (Illumina, San Diego, CA, USA). Briefly, the V3–V4 regions of the 16S rRNA gene were amplified using a two-step PCR protocol with KAPA HiFi HS ReadyMix (Nippon Genetics, Tokyo, Japan) and V3–V4 region–specific primers (F341–R805). The index PCR was performed using KAPA HiFi HS ReadyMix and a Nextera XT index kit (Illumina). The libraries were cleaned using Agencourt AMPure XP (Beckman Coulter, Brea, MA, USA) and quantified on a Qubit 3 device (Thermo Fisher Scientific, Waltham, MA, USA). The library was diluted to 8 pM (final concentration), mixed with PhiX (Illumina), and then applied to an Illumina MiSeq system for sequencing with a MiSeq reagent kit v3 (600 cycles, Illumina). The data were analyzed using the MiSeq Reporter Metagenomics Workflow (Illumina). An average of 281,476 raw reads were obtained per sample and an average of 278,461 trimmed reads per sample.

### Quantitative analysis by real-time PCR

The bacterial levels were quantitated using real-time PCR with primer pairs specific for *P. gingivalis*, *P. intermedia*, *A. actinomycetemcomitans,* and the genus *Bifidobacterium* 16S rRNA [15–18]. The sequences of the primers used for real-time PCR in this study are shown in Table 1. Real-time PCR was performed using a Light Cycler Nano Real-Time PCR system (Roche Diagnostics, Basel, Switzerland). The amplification program consisted of one cycle of 94 °C for 10 min and then 45 cycles of 95 °C for 15 s and of 60 °C for 1 min. The fluorescent data were analyzed with the Light Cycler Nano software. The number of bacteria in the saliva was represented by the logarithm to base 10 of the estimated number of cells.

**Table 1:** Primer sequences used in real-time PCR

Bacteria (16S rRNA)	Primer sequences (5′-3′)	Reference
*Bifidobacterium*		
F	CTCCTGGAAACGGGTGG	[17]
R	GGTGTTCTTCCCGATATCTACA	
*B. adolescentis*		
F	GGATCGGCTGGAGCTTGCTCCG	[19]
R	CCCCGAAGGCTTGCTCCCAGT	
P	[FAM]CTCCAGTTGGATGCATGTCCTTCTGG[TAM]	
*B. dentium*		
F	ATCCCGGGGGTTCGCCTCC	[19]
R	ATACCGATGGAACCTTTCCCGG	
P	[FAM]TGCTCCGGTTGGATGCATGTCCTTCC[TAM]	
*P. gingivalis*		
F	AGGCAGCTTGCCATACTGCG	[15]
R	ACTGTTAGCAACTACCGATGT	
*P. intermedia*		
F	TTTGTTGGGGAGTAAAGCGGG	[18]
R	TCAACATCTCTGTATCCTGCGT	
*A. actinomycetemcomitans*		
F	CTTACCTACTCTTGACATCCGAA	[16]
R	ATGCAGCACCTGTCTCAAAGC	

*PCR* polymerase chain reaction

### *Bifidobacterium* species-specific real-time PCR assays using TaqMan probe

A universal probe/primer set for *Bifidobacterium adolescentis* (*B. adolescentis*) and *Bifidobacterium dentium* (*B. dentium*) was used as described previously (Table 1) [19]. The probes and primers were synthesized by Eurofins Genomics (Tokyo, Japan). The oligonucleotide probes were labeled with FAM at the 5′ end and TAMRA at the 3′ end. Real-time PCR was carried out using a Light Cycler 96 (Roche Diagnostics). Each PCR reaction was performed in a total volume of 20 μl containing 10 μl of FastStart Essential DNA Probes Master (Roche Diagnostics), 0.5 μl each of the forward and reverse primers (final concentration, 500 nm each), an appropriate amount of the TaqMan probe (final concentration 500 nm), 1.0 μl of template DNA solution, and an appropriate amount of sterilized DNase-RNase-free water. Each amplification reaction was performed in the Light Cycler with the cycling parameters set at initial denaturation at 95 °C for 10 min and 45 cycles at 95 °C for 10 s and 58 °C for 90 s. For the standard curves, the results obtained (Cq values) for each species or group were plotted against the initial number of cells in the corresponding culture.

### Statistical analysis

16S rRNA sequencing data were analyzed using the Quantitative Insights into Microbial Ecology2 software package (QIIME2 v2019.4.0) [20] against the 16S rRNA gene sequences assigned by the Greengenes database v13.8 [21]. Analysis of the amplicon sequence data employed the DADA2 pipeline. Alpha diversity was estimated using the observed identified operational taxonomic units (OTUs), the Chao 1 diversity index, and the Shannon diversity index. The sequencing depth was determined to be 28,352 reads from alpha rarefaction. Beta diversity was evaluated based on UniFrac distances representing the fraction of the branch length of the phylogenetic tree shared between the groups. Three-dimensional principal coordinate analysis (PCoA) was performed to generate UniFrac scatterplots to visually compare microbial compositions across groups. Differences in bacterial communities between non-pregnant and pregnant women were analyzed using the weighted and unweighted UniFrac distance metric. Permutational multivariate analysis of variance (PERMANOVA) was used on the weighted and unweighted UniFrac distance matrix to determine significant differences with Bonferroni correction in microbial communities between the different groups [22]. Statistical significance was set at *p* < 0.05. Significant differences in microbial taxa abundance between non-pregnant and pregnant women were analyzed using the ANCOM tool in QIIME2 [23]. The final significance was expressed as the empirical distribution of W. To identify possible biomarkers associated with each group, a linear discriminant effect size (LEfSe) analysis was performed using the Galaxy web application (http://huttenhower.sph.harvard.edu/galaxy/). Bacterial abundance profiles were calculated at taxonomic levels from phylum to genus in percent abundance; alpha values > 0.05 and a logarithmic linear discriminant analysis (LDA) score > 2.0 were used as thresholds [24]. The differences in clinical parameters between non-pregnant and pregnant women were analyzed through the Mann–Whitney *U*-test. The correlations between clinical parameters and the number of bacteria were analyzed with Spearman’s correlation test and multiple regression analysis. The Mann–Whitney *U*-test, Spearman’s correlation test, and multiple regression analysis were performed with the statistical software package SPSS Statistics version 26 (IBM, Armonk, NY, USA). A statistical significance level of 5% (*p* < 0.05) was applied for all statistical tests.

## Results

### Clinical parameters

All pregnant women had a term delivery without complications, and all but two of the infants’ birth weights were within the normal range (mean 3018.39 g, range 2120–3775 g). The mean PPD, GI, and PESA were significantly higher in pregnant women than in non-pregnant women. However, no other clinical parameters were significantly different between non-pregnant and pregnant women. The estradiol and progesterone levels were significantly higher in pregnant women than in non-pregnant women (Table 2).

**Table 2:** Clinical parameters of non-pregnant and pregnant women

	Non-pregnant women (n = 30)	Pregnant women (n = 35)	*P*
Age (years)	31.3 ± 5.8	30.1 ± 3.9	0.649
	(20–42)	(23–40)	
Height (cm)	158.5 ± 5.9	158.4 ± 6.0	0.974
	(148.0–168.0)	(145.0–169.0)	
Weight (Kg)	53.0 ± 7.9	62.3 ± 8.4	**0.000**
	(44.0–86.0)	(48.6–80.6)	
Estradiol (pg/mL)	1.59 ± 0.12	56.04 ± 6.61	**0.000**
	(0.54–5.14)	(3.37–267.00)	
Progesterone (pg/mL)	87.60 ± 7.99	1760.85 ± 106.91	**0.000**
	(3.37–394.09)	(283.42–9187.01)	
Infant birth weight (g)	–	3018.39 ± 425.42	–
		(2120–3775)	
Mean PPD (mm)	1.96 ± 0.17	2.22 ± 0.28	**0.000**
	(1.7–2.3)	(1.6–2.9)	
PD ≥ 4 mm (%)	0.33 ± 0.83	1.48 ± 3.59	0.647
	(0–4.17)	(0–10.71)	
BOP (%)	8.18 ± 6.25	7.77 ± 8.15	0.427
	(0–20.8)	(0–28.6)	
Mean GI	0.04 ± 0.08	0.29 ± 0.41	**0.001**
	(0–0.30)	(0–1.43)	
PESA (mm^2^)	968.97 ± 106.86	1199.29 ± 155.30	**0.000**
	(780.5–1166.3)	(891.6–1571.2)	
PISA (mm^2^)	86.74 ± 71.02	106.57 ± 122.13	0.946
	(0–237.4)	(0–521)	
DMF	6.03 ± 5.71	5.59 ± 5.03	0.780
	(0–22)	(0–17)	
Smoker	3	3	0.844
	(former 1, current 2)	(former 3)	

Bold values are statistically significant

Clinical cut offs; probing depth ≥ 4 mm and ≥ 10% BOP (Definition of healthy; no probing attachment loss, probing depth ≤ 3 mm and ≤ 10% BOP)

Values are shown as mean ± standard deviation (SD) (range) or percentage ± SD (range)

*PPD* probing pocket depth, *BOP* bleeding on probing, *GI* gingival index, *PESA* periodontal epithelial surface area, *PISA* periodontal inflamed surface area, *DMF* decayed, missing, and filled teeth. Statistical analysis of clinical parameters in non-pregnant and pregnant women was performed by Mann–Whitney *U*-test

### Alpha and beta diversity analysis

**Fig. 1:**
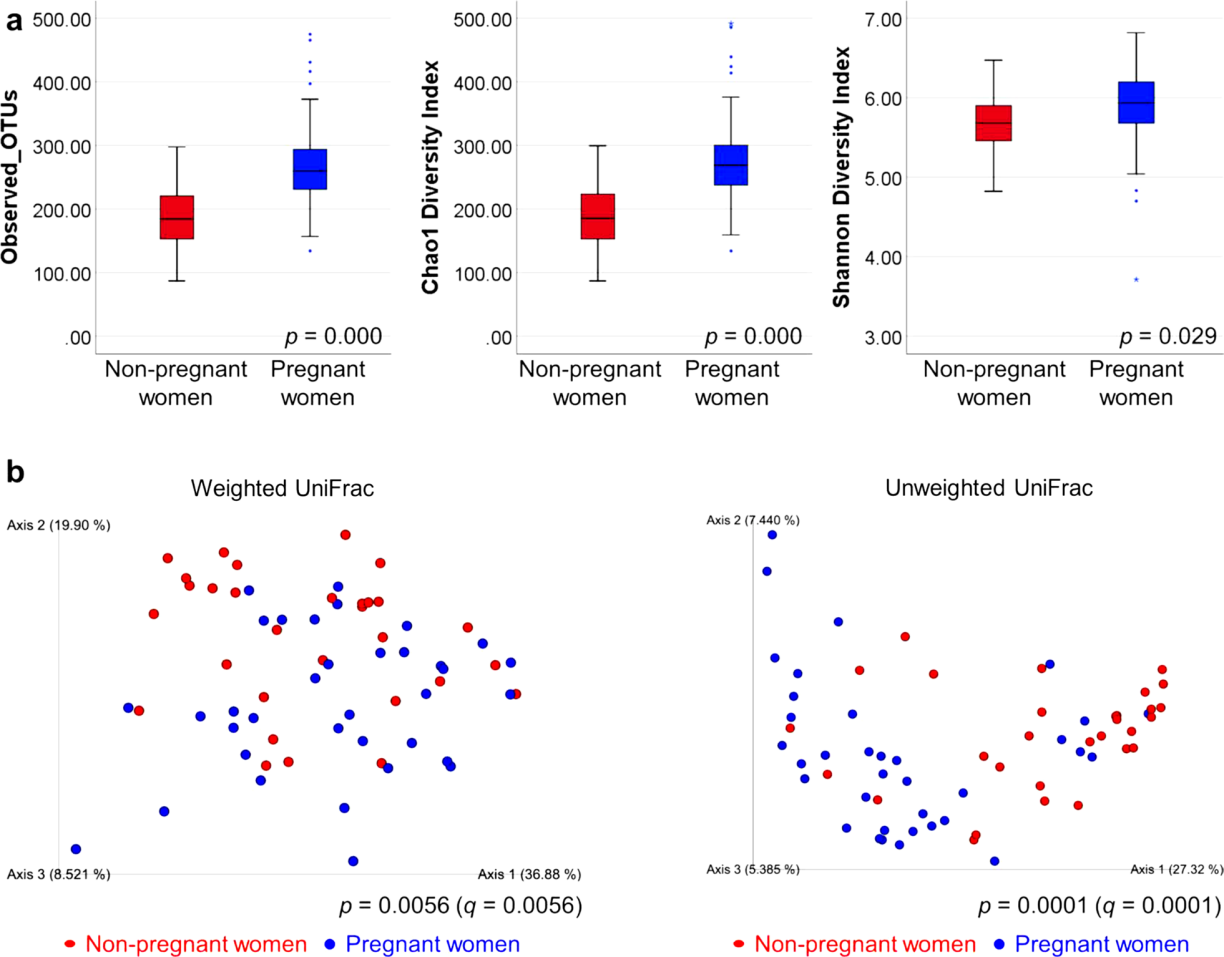
Comparison of microbial diversity in the saliva in non-pregnant and pregnant women. **a** Rarefaction analysis of 16S rRNA gene sequences obtained from non-pregnant women and pregnant women. **b** PCoA representing the beta diversity estimated from the weighted and unweighted UniFrac distances of 16S rRNA genes in non-pregnant women and pregnant women

All 65 samples were sequenced using MiSeq, and 6,702,139 total sequences were amplified (range, 28,352–185,102 sequences per sample; mean, 103,110 sequences per sample). The taxonomic identity of the reads was analyzed using QIIME2. The observed OTUs, Chao1 diversity index, and Shannon diversity index were significantly higher in pregnant women than in non-pregnant women (Fig. 1a). Additionally, weighted and unweighted UniFrac distances were significantly different between the microbial communities of non-pregnant and pregnant women (Fig. 1b).

### Oral bacterial taxonomy of the saliva

**Fig. 2:**
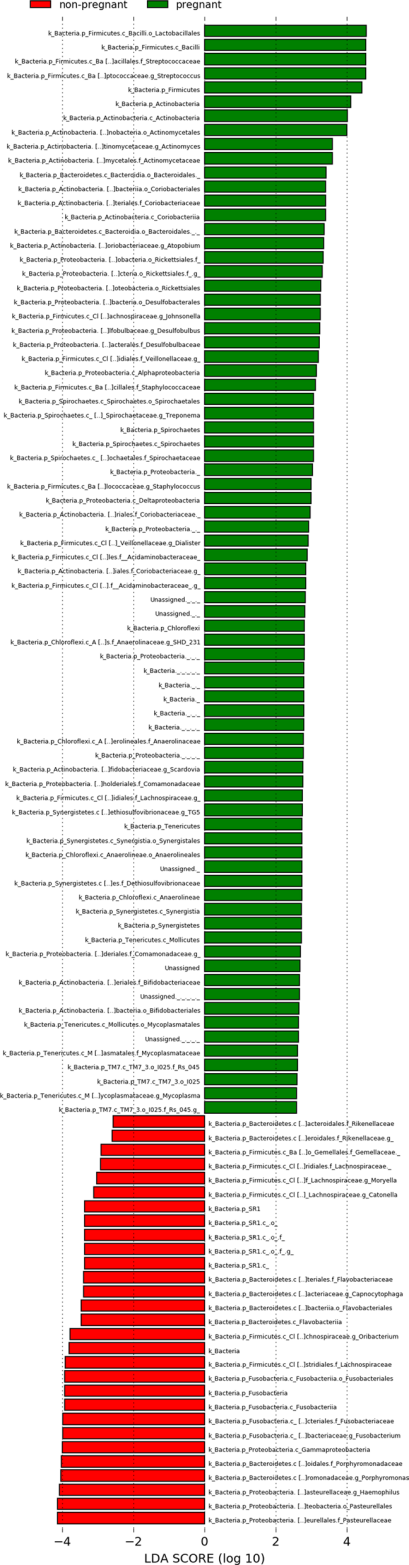
Linear discriminant analysis effect size. The differentially abundant taxonomic profile of saliva microbiota of pregnant women versus non-pregnant women

QIIME2 helped detect a total of 167 different bacterial genera in non-pregnant and pregnant women. The most abundant genus among all samples was *Prevotella*, followed by *Streptococcus*, *Veillonella*, and *Neisseria*. We analyzed the bacterial DNA sequence profiles of the saliva samples and identified significant differences in the microbial taxa between non-pregnant and pregnant women using the ANCOM feature of the QIIME2 program. At the order level, *Bifidobacteriales, Lactobacillales, Actinomycetales,* and *Mycoplasmatales* were determined to be differentially abundant between non-pregnant and pregnant women (Table 3). The order with the most significant difference between the two groups was *Bifidobacteriales* (W = 21). *Bifidobacteriales* had the largest effect size difference in pregnant women compared with non-pregnant women (median = 18.0, max = 1143.0). At the family level, *Bifidobacteriaceae, Streptococcaceae*, and *Carnobacteriaceae* were determined to be differentially abundant between non-pregnant and pregnant women (Table 3). The most significant difference was observed in *Bifidobacteriaceae* levels (W = 30). *Bifidobacteriaceae* had the largest effect size difference in pregnant women compared with non-pregnant women (median = 18.0, max = 1143.0). No significant differences were observed at the genus level between non-pregnant and pregnant women using the ANCOM method. To evaluate microbial contents between non-pregnant and pregnant women, LDA was performed using LEfSe. We identified 106 bacterial taxa with LDA > 2.0. There were 77 bacterial taxa in the pregnant women with the highest LDA score. They belonged to the family *Bifidobacteriaceae* (phylum *Actinobacteria*, class *Actinobacteria*, order *Bifidobacteriales*) and order *Bifidobacteriales* (phylum *Actinobacteria*, class *Actinobacteria*), consistent with the ANCOM findings. There were 29 bacterial taxa in the non-pregnant women with the highest LDA score (Fig. 2).

**Table 3:** Significant differential abundance of the genera with their 50th percentile abundance, max percentile abundance, and W-statics of the ANCOM method

	Median percentile abundance		Max percentile abundance		W
	Non-pregnant	Pregnant	Non-pregnant	Pregnant	
*Order*					
*Bifidobacteriales*	1.0	18.0	98.0	1143.0	21
*Lactobacillales*	6919.0	25,800.0	28,490.0	68,006.0	15
*Actinomycetales*	2258.0	7289.0	10,058.0	23,072.0	11
*Mycoplasmatales*	1.0	12.0	133.0	513.0	11
*Family*					
*Bifidobacteriaceae*	1.0	18.0	98.0	1143.0	30
*Streptococcaceae*	5849.5	23,121.0	25,192.0	65,374.0	15
*Carnobacteriaceae*	814.0	2284.0	3257.0	4667.0	11

ANCOM was performed to identify significant differences in order abundances based on 50th percentile abundance (Median), highest sequence count found in a sample (Max), and W statistic

### Real time PCR analysis of *Bifidobacterium* and periodontopathic bacteria

**Fig. 3:**
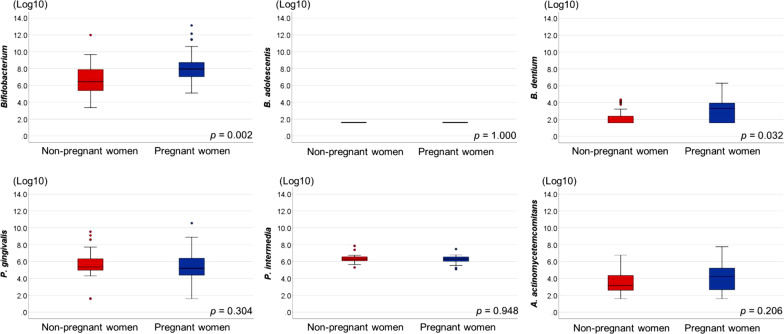
Oral bacteria in non-pregnant and pregnant women as analyzed by Mann–Whitney *U*-test

The genus *Bifidobacterium*, *Bifidobacterium* species (*B. adolescentis* and *B. dentium*), and periodontopathic bacteria (*P. gingivalis, P. intermedia,* and *A. actinomycetemcomitans*) were quantified using real time PCR. The genus *Bifidobacterium* and *B. dentium* numbers were significantly higher in pregnant women than in non-pregnant women. However, *B. adolescentis* was not detected in pregnant or non-pregnant women. No significant difference in the number of periodontopathic bacteria was found between pregnant and non-pregnant women (Fig. 3).

### Correlation between clinical parameters, periodontopathic bacteria, and *Bifidobacteria*

The results of the Spearman’s correlation test between clinical parameters and oral bacteria in all subjects (non-pregnant and pregnant women) are shown in Table 4. The abundance of the genus *Bifidobacteria* significantly correlated with patient weight (*p* = 0.013), mean GI (*p* = 0.00004), DMF (*p* = 0.021) and estradiol levels (*p* = 0.0004). *Bifidobacterium* *dentium* abundance was significantly correlated with the mean PPD (*p* = 0.009), PESA (*p* = 0.007), estradiol (*p* = 0.037), and progesterone (*p* = 0.006) levels.

**Table 4:** Spearman’s correlation coefficients between clinical parameters and oral bacteria in non-pregnant and pregnant women

	*Bifidobacterium*		*B. dentium*		*P. gingivalis*		*P. intermedia*		*A. actinomycetemcomitans*	
	All subjects	Pregnant women	All subjects	Pregnant women	All subjects	Pregnant women	All subjects	Pregnant women	All subjects	Pregnant women
*Age*										
Rho	− 0.125	− 0.471	0.138	0.223	0.476	0.475	− 0.086	0.078	− 0.168	− 0.155
*p*	0.320	**0.004**	0.272	0.199	**0.000**	**0.004**	0.498	0.656	0.180	0.374
*Height*										
Rho	0.048	− 0.117	− 0.174	− 0.302	0.029	− 0.021	− 0.023	0.018	− 0.009	0.114
*p*	0.707	0.504	0.166	0.078	0.816	0.903	0.855	0.918	0.941	0.513
*Weight*										
Rho	0.307	0.068	0.102	− 0.047	− 0.078	− 0.026	− 0.100	− 0.087	− 0.099	− 0.348
*p*	**0.013**	0.700	0.419	0.789	0.538	0.884	0.428	0.620	0.432	**0.040**
*Estradiol*										
Rho	0.424	0.281	0.259	0.100	− 0.203	− 0.413	− 0.098	− 0.268	0.101	− 0.009
*p*	**0.000**	0.103	**0.037**	0.569	0.106	0.014	0.439	0.120	0.421	0.959
*Progesterone*										
Rho	0.135	− 0.208	0.339	0.269	− 0.089	− 0.195	0.044	0.010	0.221	0.026
*p*	0.282	0.231	**0.006**	0.119	0.480	0.262	0.729	0.955	0.076	0.882
*Mean PPD*										
Rho	0.108	− 0.015	0.322	0.133	− 0.025	− 0.108	− 0.071	− 0.236	0.139	− 0.112
*p*	0.395	0.933	**0.009**	0.452	0.844	0.545	0.576	0.180	0.273	0.527
*BOP*										
Rho	− 0.072	0.244	0.146	0.089	− 0.166	− 0.209	0.089	− 0.090	0.049	− 0.092
*p*	0.570	0.165	0.248	0.618	0.190	0.237	0.483	0.613	0.701	0.604
*Mean GI*										
Rho	0.489	0.431	0.131	0.029	− 0.284	− 0.482	− 0.193	− 0.179	− 0.230	− 0.437
*p*	**0.000**	**0.011**	0.303	0.870	**0.023**	**0.004**	0.126	0.312	0.068	**0.010**
*PESA*										
Rho	0.233	0.203	0.331	0.153	− 0.134	− 0.284	0.004	− 0.213	0.154	− 0.192
*p*	0.064	0.250	**0.007**	0.389	0.290	0.104	0.976	0.227	0.223	0.278
*PISA*										
Rho	− 0.035	0.221	0.194	0.042	− 0.182	− 0.260	0.127	− 0.028	0.079	− 0.097
*p*	0.781	0.210	0.126	0.813	0.150	0.138	0.318	0.877	0.534	0.587
*DMF*										
Rho	0.287	0.180	0.125	0.253	− 0.159	− 0.178	− 0.349	− 0.316	− 0.204	0.143
*p*	**0.021**	0.309	0.327	0.149	0.210	0.313	**0.005**	0.069	0.107	0.421

Bold values are statistically significant

*Porphyromonas* *gingivalis* abundance was significantly positively correlated with age (*p* = 0.00006) and significantly negatively correlated with mean GI (*p* = 0.023). *Prevotella* *intermedia* abundance was significantly negatively correlated with DMF (*p* = 0.005).

The genus *Bifidobacteria* abundance was significantly positively correlated with the mean GI (*p* = 0.011) and significantly negatively correlated with age (*p* = 0.004). *Porphyromonas* *gingivalis* abundance was significantly positively correlated with age (*p* = 0.004) and significantly negatively correlated with mean GI (*p* = 0.004). *Aggregatibacter* *actinomycetemcomitans* abundance was significantly negatively correlated with weight (*p* = 0.04) and mean GI (*p* = 0.01).

The Spearman’s correlation coefficients between clinical parameters and salivary hormones are shown in Table 5. Both estradiol and progesterone were significantly associated with weight, mean PPD, mean GI, and PESA in all subjects (Table 5).

**Table 5:** Spearman’s correlation coefficients between clinical parameters and salivary hormones

	Estradiol		Progesterone	
	All subjects	Pregnant women	All subjects	Pregnant women
*Age*				
Rho	− 0.146	− 0.363	− 0.057	0.157
*p*	0.247	**0.032**	0.652	0.368
*Height*				
Rho	0.001	− 0.085	− 0.083	− 0.120
*p*	0.992	0.629	0.510	0.491
*Weight*				
Rho	0.647	0.287	0.490	− 0.005
*p*	**0.000**	0.095	**0.000**	0.978
*Mean PPD*				
Rho	0.498	0.168	0.562	0.168
*p*	**0.000**	0.342	**0.000**	0.341
*PPD* ≥ *4 mm*				
Rho	0.076	0.170	0.114	− 0.057
*p*	0.551	0.337	0.369	0.748
*BOP*				
Rho	0.009	0.221	− 0.029	− 0.053
*p*	0.941	0.209	0.820	0.765
*Mean GI*				
Rho	0.530	0.356	0.438	0.164
*p*	**0.000**	**0.039**	**0.000**	0.353
*PESA*				
Rho	0.662	0.308	0.700	0.191
*p*	**0.000**	0.076	**0.000**	0.280
*PISA*				
Rho	0.096	0.204	0.096	− 0.021
*p*	0.452	0.246	0.079	0.908
*DMF*				
Rho	0.040	0.217	− 0.088	0.174
*p*	0.756	0.218	0.491	0.326

Bold values are statistically significant

Furthermore, the genus *Bifidobacteria* abundance was negatively associated with *P. gingivalis* and *A. actinomycetemcomitans* abundance in pregnant women as well as in all subjects (Table 6).

**Table 6:** Spearman’s correlation coefficients between *Bifidobacterium* and periodontopathic bacteria

	*Bifidobacterium*		*B. dentium*	
	All subjects	Pregnant women	All subjects	Pregnant women
*P. gingivalis*				
Rho	− 0.295	− 0.462	0.027	− 0.024
*p*	**0.017**	**0.005**	0.830	0.892
*P. intermedia*				
Rho	− 0.111	− 0.042	0.178	0.107
*p*	0.378	0.809	0.156	0.539
*A. actinomycetemcomitans*				
Rho	− 0.338	− 0.366	0.025	− 0.204
*p*	**0.006**	**0.031**	0.843	0.239

Bold values are statistically significant

### Multivariate analysis

The results from the multiple regression analysis are shown in Table 7.* Bifidobacterium* *dentium* abundance was significantly associated with the concentration of salivary progesterone in all subjects. The relationship was also observed in pregnant women (Table 7).

**Table 7:** Multiple regression analysis of the association between salivary *B. dentium* and other parameters

Model	Unstandardized coefficient		Standardized coefficient	t	*p*	95% Cl		Adjusted R^2^
	(β)	Standard error	(β)			Low	High	
*All subjects*								
(Constant)	2.178	0.178		12.236	0.000	1.822	2.534	0.198
Progesterone	0.000	0.000	0.445	3.916	0.000	0.000	0.001	
*Pregnant women*								
(Constant)	2.243	0.338		6.638	0.000	1.555	2.931	0.159
Progesterone	0.000	0.000	0.429	2.686	0.011	0.000	0.001	

The dependent variable was *B. dentium* abundance. Independent variables were age, height, weight, mean PPD, %PPD ≥ 4 mm, %BOP, mean GI, DMF, smoking, estradiol, progesterone, *P. gingivalis* abundance, *P. intermedia* abundance, and *A. actinomycetemcomitans* abundance.* CI* confidence interval

## Discussion

The present study examined the association between salivary bacteria and female hormones. Unstimulated saliva has been reported to be optimal for the determination of female hormones. At the family level, *Bifidobacteriaceae*, *Streptococcae,* and *Carnobacteriaceae* were increased in pregnant women. Among them, W of *Bifidobacteriaceae* was the highest. The results of the present study indicate that *Bifidobacteria* abundance increased parallel to the increasing estradiol and progesterone concentrations (Tables 4 and 7). In accordance with this finding, estradiol and progesterone support the growth of *Bifidobacterium* spp. [25], and progesterone promotes *Bifidobacterium* growth in the gut microbiota during pregnancy [26]. Thus, the increased levels of *Bifidobacteria* observed in the saliva of pregnant women could be explained by the increase in these hormones.

The alpha and beta diversities were higher in the samples from pregnant women than in those from non-pregnant women. *Bifidobacteriales* abundance was higher in pregnant women than in non-pregnant women (Fig. 1 and Table 3).

Pregnant women experience gingival inflammation and bleeding and exhibit deeper PPDs than non-pregnant women [6]. In this study, the PPD and mean GI of pregnant women were slightly greater than those of non-pregnant women (Table 2). Furthermore, the PPD and GI values were associated with both estradiol and progesterone salivary concentrations. In fact, pregnancy-associated gingivitis improves postpartum [27]. Generally, plasma estradiol and progesterone levels are 10 and 30 times higher, respectively, in pregnant women than in menstruating women [6]. Salivary estradiol and progesterone levels are also higher in pregnant women than in non-pregnant women [28–31]. These hormones are used as nutrients by bacteria, such as *P. intermedia* [28] and *P. gingivalis* [32]. Several studies have reported that increased levels of *P. intermedia* in pregnant women are associated with gingivitis [2, 3, 6], and another study suggested that *P. gingivalis* significantly contributes to the worsening of gingival inflammation during pregnancy [4]. In addition to the bacterial factor, estradiol and progesterone enhanced the production of inflammatory cytokines by human gingival fibroblasts, suggesting that female sex hormones enhance gingivitis during pregnancy [33].

Inflammation may be a risk factor for preterm birth and for morbidity in preterm infants. Periodontitis is a chronic inflammatory disease caused by bacterial plaque attached to the tooth surface. *Porphyromonas* *gingivalis* is a late colonizer in the plaque biofilm [34] and is associated with local and systemic inflammation in patients with periodontitis [35]. The presence of *P. gingivalis* in pregnant women is associated with an increased risk of preterm delivery and low infant birth weight [7], and appropriate periodontal treatment might potentially be associated with suppression of preterm birth [12, 36–41].

In this study, estradiol and progesterone levels were increased in the saliva of pregnant women (Table 2), but the *P. intermedia* and *P. gingivalis* levels were not significantly different between pregnant and non-pregnant women (Fig. 2). The increased levels of *Bifidobacteria* observed in healthy pregnant women could suppress *P. intermedia* because both *Bifidobacterium* and *P. intermedia* use these hormones as nutrients. Lin et al. reported that *Neisseria*, *Porphyromonas,* and *Treponema* were more abundant in the salivary microbiota of pregnant women compared with non-pregnant women [11]. The GI reported in their study was higher than that observed in the present study, and the relationship between BOP and GI may vary according to PPD at the individual site examined [42], suggesting that high GI may be associated with plaque-associated gingival inflammation. GI is slightly increased in healthy women throughout pregnancy without a concomitant increase in the plaque levels [43]. The lack of increase in periodontopathic bacteria and slightly increased GI values observed in the current study might be attributed to the participants, who were periodontally healthy and had minimal plaque associated-gingivitis. Consistent with this, the GI negatively correlated with *P. gingivalis* in this study.

*Bifidobacterium* is a major symbiotic intestinal microbe in infants [44, 45]. *Bifidobacterium* seems to play a crucial role in protecting against pathogens, contributing to the priming of the mucosal immune system and maintaining human health [46–48]. In this study, the genus *Bifidobacterium* abundance was correlated with DMF, but the association was not observed with *B. dentium.* Several reports have suggested that *B. dentium* is associated with the development of plaque and dental caries [49–51]. *Bifidobacterium* *dentium* is frequently isolated from children and young adolescents with severe caries [52] and is related to fluoride tolerance [53]. Although *Bifidobacterium* is considered to be a symbiotic bacterium, the presence of *Bifidobacterium* may be a risk factor for caries development.

*Bifidobacterium* *dentium* is, however, well-adapted for commensalism in the gastrointestinal tract [54]. Hojo et al. reported that the *B. dentium* count may be associated with periodontal health [55]. *Bifidobacteria* were shown to inhibit the growth of *P. gingivalis* in an in vitro biofilm model [56]. *Bifidobacterium* strains are nutritionally competitive with *P. gingivalis*, and consumption of vitamin K by *P. gingivalis* may suppress *Bifidobacterium* strains [32]. *Bifidobacterium* may interfere with the growth of *P. gingivalis* through nutrient and niche deprivation, and suppression of *P. gingivalis* is important in increasing symbiotic *Bifidobacterium* in the mother’s saliva. In the present study, the genus *Bifidobacterium* abundance was negatively correlated with *P. gingivalis*, but this relationship was not observed with *B. dentium*. Furthermore, probiotic therapy using *Bifidobacterium* improved clinical parameters in chronic periodontitis [57]. *Bifidobacterium* suppressed biofilm formation and transcription of pathogenic genes of *P. gingivalis* [58]. A limitation of this study is its cross-sectional study design; we could not discuss the causal relationships between *Bifidobacterium* and healthy pregnancy. In addition, although we found that *Bifidobacterium* was significantly increased in the saliva of pregnant women, we could not determine all *Bifidobacterium* species.

## Conclusion

Within the limitations of this study, our investigation showed that the abundance of the genus *Bifidobacterium* and particularly of *B. dentium* was greater in pregnant women than in non-pregnant women.

## Data Availability

The data have been deposited with links to BioProject accession number PRJDB9791 in the DDBJ BioProject database.

## Abbreviations

ANCOM: Analysis of composition of microbiome
BOP: Bleeding on probing
DMF: Decayed, missing, and filled teeth
ELISA: Enzyme-linked immunosorbent assay
GI: Gingival index
LDA: Linear discriminant analysis
LEfSe: Linear discriminant effect size
OUT: Operational taxonomic unit
PCoA: Principal coordinate analysis
PERMANOVA: Permutational multivariate analysis of variance
PESA: Periodontal epithelial surface area
PISA: Periodontal inflamed surface area
PPD: Probing pocket depth
